# Supplementary material for: Modelling Skylarks (Alauda arvensis) to Predict Impacts of Changes in Land Management and Policy: Development and Testing of an Agent-Based Model
Source: PLoS One. 2013 Jun 6;8(6):e65803. doi: 10.1371/journal.pone.0065803 (PMC3675089; doi:10.1371/journal.pone.0065803)
Supplement: Supporting Information S4 — The skylark ODdox as a zipped archive. (ZIP) [file pone.0065803.s004.zip › Skylark_ODdox/functions_0x6e.html]

ALMaSS Skylark ODdox: Class Members


ALMaSS Skylark ODdox
 2.0


- Main Page
- Related Pages
- Classes
- Files

- Class List
- Class Index
- Class Hierarchy
- Class Members

- All
- Functions
- Variables
- Typedefs

- a
- b
- c
- d
- e
- f
- g
- h
- i
- j
- k
- l
- m
- n
- o
- p
- r
- s
- t
- u
- v
- w
- x
- y
- z
- ~

Here is a list of all class members with links to the classes they belong to:

### - n -

- n
  : IntArray100
- NaturalGrass()
  : NaturalGrass
- NestLoc
  : Skylark\_Female
- NewCrop
  : Rotation
- NewElement()
  : Landscape
- No
  : skClutch\_struct
- No\_HabitatTable\_Refs
  : Skylark\_Male
- NonVegElement()
  : NonVegElement
- NoPesticideBaseFarm()
  : NoPesticideBaseFarm
- NoPesticideNoPFarm()
  : NoPesticideNoPFarm
- NoTerritories
  : SkTerritories
- nqual
  : skTerritory\_struct
- NWordOutput()
  : Population\_Manager
- NWordOutputPrb
  : Population\_Manager


- Generated on Thu Jan 10 2013 13:15:39 for ALMaSS Skylark ODdox by
   1.8.1.1
